# Acceptance, Knowledge, and Attitude of Parents Toward the Human Papillomavirus Vaccine in the Eastern Region of Saudi Arabia: A Cross-Sectional Study

**DOI:** 10.7759/cureus.51293

**Published:** 2023-12-29

**Authors:** Latteefah Alnaeem, Shatha Alanizi, Ghaida AlQarni, Jana Alwadani, Fatimah Bomouzah, Zainab Ali

**Affiliations:** 1 Obstetrics and Gynecology, King Faisal University, Al-Ahsa, SAU; 2 Medical School, King Faisal University, Al-Ahsa, SAU; 3 Medical School, King Faisal University, Al-Hofuf, SAU; 4 College of Medicine, King Faisal University, Al-Ahsa, SAU

**Keywords:** saudi arabia, knowledge and awareness, human papillomavirus, cervical cancer, human papillomavirus vaccine

## Abstract

Background

Human papillomavirus (HPV) is a common sexually transmitted virus associated with conditions such as skin warts and cervical cancer. Although many individuals clear the infection, some face persistent risks. Cervical cancer, which is linked to certain types of HPV, is a major health concern both globally and in Saudi Arabia, with preventive measures including HPV vaccination. However, parental knowledge and attitudes toward vaccinating their children vary. Therefore, this research aims to assess parental acceptance and understanding of the HPV vaccine in the Eastern Region of Saudi Arabia.

Methodology

This cross-sectional study was conducted in the Eastern Region of Saudi Arabia using an online questionnaire during 2022-2023. The data were cleaned in Excel and analyzed using SPSS version 29 (IBM Corp., Armonk, NY, USA). The study assessed parents’ knowledge and acceptance of HPV vaccination.

Results

A total of 380 participants were included in this study, the majority of whom were female, married, well-educated, and residents of Al-Ahsa, Saudi Arabia. Awareness about the HPV vaccine was modest, with only 46.6% of participants having heard of it. Most parents reported that their doctors did not mention the vaccine (62.9%), and 67.1% stated that their children had not received it. Factors influencing acceptance included support from the Ministry of Health and belief in the vaccine’s effectiveness. Concerns about side effects and vaccine effectiveness were the main barriers to vaccination. Sociodemographic factors, including gender, age, education, employment, and number of children, significantly influenced both knowledge and acceptance. Notably, awareness of HPV was strongly associated with acceptance.

Conclusions

Our study revealed limited knowledge and vaccine acceptance among parents in the Eastern Region of Saudi Arabia. Sociodemographic factors, including gender, age, education, employment, and number of children, significantly influenced both knowledge and acceptance. Thus, sociodemographic factors played a significant role in shaping these attitudes, emphasizing the need for targeted awareness campaigns and improved communication between healthcare providers and parents to enhance vaccine uptake.

## Introduction

Human papillomavirus (HPV) is a double-stranded DNA virus and one of the most common sexually transmitted infections. It is mainly transmitted through sexual contact and infects the cutaneous and mucosal epithelium, which are associated with common skin warts and cervical cancer, respectively. Although most infected individuals will eventually clear the infection, a persistent infection remains a risk for all affected individuals [1]. The HPV family has over 200 genotypes, with types 16 and 18 being the cause of approximately 70% of cervical cancer cases. HPV has been also associated with anal, vaginal, valvular, penile, and oropharyngeal cancer [2].

Cervical cancer is the fourth most common cancer in females worldwide and the eighth most common cancer among females in Saudi Arabia. In 2020, it was estimated that approximately 358 cases are diagnosed with cervical cancer annually in Saudi Arabia. The annual number of deaths due to cervical cancer in Saudi Arabia is approximately 179, making it the seventh leading cause of cancer deaths in women 15-44 years of age [2].

HPV-related cancers can be prevented using primary prevention, which includes HPV vaccination [3]. In 2006, the U.S Food and Drug Administration approved the quadrivalent HPV vaccine (Gardasil), which protects against HPV6, 11, 16, and 18, to be used in females 9-26 years of age; by 2017, 71 countries worldwide introduced the HPV vaccine in their national vaccination programs for young girls [4]. In Saudi Arabia, the vaccine was first approved by the Saudi Food and Drug Administration in 2010 and introduced in the updated national immunization schedule in 2019 for girls 11-12 years of age [5,6].

In 2020, the World Health Organization (WHO) adopted a global strategy to eliminate cervical cancer, marking the first global health strategy to eliminate a type of cancer. The strategy proposes that, by 2030, each country should reach a target of 90% of girls being fully vaccinated against HPV by 15 years old [3]. Because the HPV vaccine is better administered before exposure to HPV through sexual contact, WHO recommends vaccination of girls at 9-14 years of age [7]. In line with WHO’s global strategy to eliminate cervical cancer, Saudi Arabia has launched a national vaccination program to vaccinate girls 9-13 years of age by visiting several schools to provide the vaccine [8,9]. The vaccination has been also provided in several hospitals and primary health centers, where the individual must visit the location to receive the vaccine. Parents’ awareness and acceptance of the HPV vaccine are required for them to take their daughters to a healthcare center to get vaccinated.

Several studies globally have shown that parents have limited knowledge about the HPV vaccine. In Qatar, Hendaus et al. reported that >60% of parents were not aware that HPV can cause cancers such as cervical and genital cancers [10]. However, 77% of parents responded as being “very comfortable” with giving their children a vaccine that would protect them from getting genital cancer. However, <4% of parents said that their children’s doctors ever recommended the HPV vaccine [10]. In Ethiopia, a study with 638 participants showed that only 35.8% of parents were knowledgeable about HPV vaccination, and 44.8% were willing to have their children vaccinated [11].

In Saudi Arabia, only two studies have investigated parents’ attitudes and knowledge toward the HPV vaccine, which were done in Riyadh and the Western Region [12,13]. Parents’ acceptance and knowledge are determining factors in the vaccination of the younger population, but, according to our research, there have not been any study exploring this in the Eastern Region of Saudi Arabia. Therefore, our study aims to estimate the attitudes, knowledge, and acceptance of the HPV vaccine among parents in the Eastern Region of Saudi Arabia.

## Materials and methods

A cross-sectional study design was used in this investigation. A survey was undertaken online with data gathered using a questionnaire administered and distributed on different social media platforms that participants completed after providing their consent to participate in the study. The study area comprised the Eastern Region of Saudi Arabia for a duration of 11 months from November 2022 to October 2023. A total of 380 eligible participants were included in the study determined using the Raosoft Sample Size Calculator with a 95% confidence interval and a 7% margin of error. The inclusion criteria were as follows: ≥18 years old, residing in the Eastern Region of Saudi Arabia, and literate. The exclusion criteria were as follows: no children and data-entry errors. The questionnaire was entirely original, as no questionnaire that related specifically to parents’ orientation with HPV vaccination was found. Accordingly, a new questionnaire was designed by gathering some questions from previously validated and reliable questionnaires covering elements related to the awareness of HPV vaccines [10,12,14].

The questions were subjected to reliability through Cronbach’s alpha coefficient for scale data of 0.68. Permission to undertake this research was sought, and approval was granted by the ethical committee of King Faisal University in Al-Ahsa (approval number: KFU-REC-2022-NOV-ETHICS337). The participants engaged in the survey were informed that their involvement was voluntary and that completing the distributed questionnaires implied that they had agreed to participate in the study. The questionnaire was designed following a comprehensive discussion with a team of experts in the field of general obstetrics and gynecology and was then validated by a team of experts, including consultants and an associate consultant from the Obstetrics and Gynecology Department at King Faisal University. The content of the questionnaire was translated into Arabic to preserve the meaning of the important elements it captured. The translated copy of the questionnaire was authenticated in terms of its face and content validity. The final copy of the questionnaire that was used in the actual study included 29 questions grouped into four sections. The first section contained eight questions on biographical data such as age, gender, marital status, educational level, and field of study. The second section contained 11 questions to assess general knowledge about the HPV vaccine. The third section contained seven questions on awareness of HPV vaccines. The fourth section contained three questions that assessed the willingness and acceptance of parents and their partners for their children to receive the HPV vaccine. The questions had multiple response choices, with one “I don’t know” option to avoid guesses from the respondents. A score of 1 was assigned to each correct answer and 0 to each incorrect or “I don’t know” answer. A higher score indicated that the respondent had better knowledge about HPV and its vaccine. The maximum score was 18 (second and third sections), and the minimum was 0. The total knowledge score was grouped as follows: 0-6 = poor knowledge, 7-12 = fair knowledge, and 13-18 = good knowledge. The percentage of knowledge score was then calculated as the total score obtained divided by the maximum score (i.e., 18 points). Relationships between social variables and level of knowledge of HPV were measured using Fisher’s exact test, where p < 0.05 was considered statistically significant. In the fourth section, we aimed to create a list of factors that influenced parents’ decision-making and correlate that with their final level of knowledge. Data were extracted, coded, and analyzed using SPSS version 29 (IBM Corp., Armonk, NY, USA).

## Results

**Figure 1:**
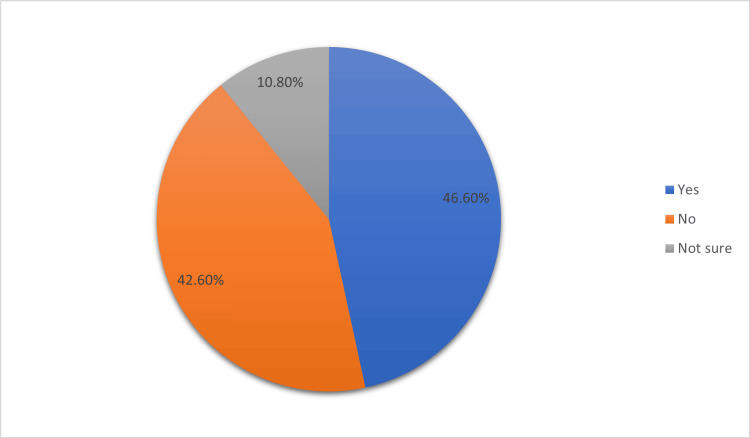
Proportion of parents who heard about human papillomavirus vaccine.

Our study included 380 participants, the majority of whom were female (62.8%), 41-50 years of age (38.7%), married (97.3%), well-educated (72.2% had a bachelor’s degree or higher), and residents of Al-Ahsa (43.1%). Most participants worked in sectors other than healthcare (91.1%), and the number of children varied, with three to four children being the most common (32.7%) (Table 1). Figure 1 shows the proportion of participants who had heard about the HPV vaccine. Overall, 46.6% of participants had heard about HPV infection, whereas 42.6% had not heard about it, and 10.8% were not sure, although knowledge levels were modest (mean score = 3.00 out of 11).

**Table 1:** Sociodemographic data of participants assessed for acceptance, knowledge, and attitude of parents toward the human papillomavirus (HPV) vaccine.

		Frequency (n = 380)	Percentage (%)
Gender	Female	239	62.8
	Male	141	37.2
Age (years)	18–30	42	11.1
	31–40	108	28.4
	41–50	147	38.7
	>50	83	21.8
Marital status	Married	370	97.3
	Widowed/Divorced	10	2.7
Residency	Al-Ahsa	167	43.1
	Dammam	81	21.5
	Dhahran	48	12.7
	Qatif	38	10.0
	Khobar	31	8.4
	Other	16	4.4
Educational status	Up to high school	106	27.8
	Bachelor’s or higher	274	72.2
Occupation	Employed	200	52.6
	Freelancer	16	4.2
	Housewife	18	4.7
	Retired	58	15.3
	Student	10	2.6
	Unemployed	78	20.6
Field of work	Healthcare sector	34	8.9
	Other sectors	346	91.1
Number of children	1–2	103	27.1
	3–4	124	32.7
	5–6	121	31.8
	>7	32	8.4
General information score about HPV (out of 11)	Mean (SD)	3.00 (2.9)	
	Range	0–9.9	

Table 2 provides insights into parents’ acceptance of the HPV vaccine. A majority of parents mentioned that their doctors did not mention the vaccine (62.9%). Regarding vaccination, 67.1% of parents reported that their children had not received it. A minority of parents agreed to have their child vaccinated by age 12 (41.1%). For spousal agreement, a notable number of parents (25.6%) reported their spouse’s agreement to HPV vaccination.

**Table 2:** Questionnaire investigating acceptance of parents toward the human papillomavirus (HPV) vaccine.

		No	Don’t know	Yes
The HPV vaccine is mentioned by your doctor	n	239	110	31
	%	62.9	28.9	8.2
Any of your children given the HPV vaccine	n	255	98	27
	%	67.1	25.7	7.2
You agree to have your child receive the HPV vaccine by age 12	n	224	0	156
	%	58.9	0.0	41.1
Your spouse agrees to have your child receive the HPV vaccine by age 12	n	132	151	97
	%	34.7	39.7	25.6

**Figure 2:**
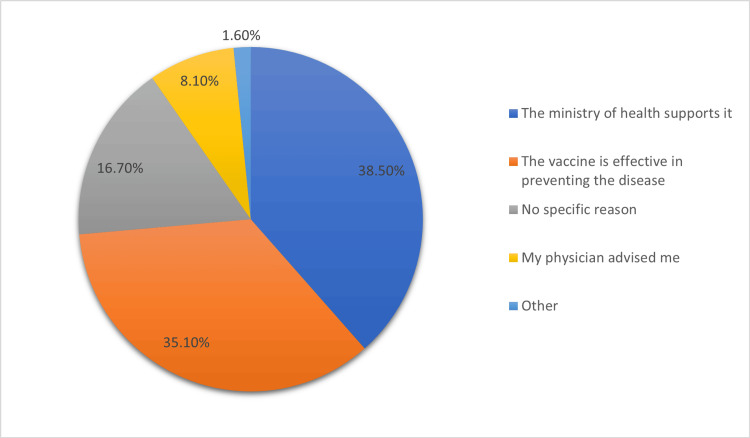
Factors influencing parents’ decision to vaccinate their child against human papillomavirus.

Figure 2 shows the factors influencing parents’ decision to have their child vaccinated against HPV. Overall, 38.5% of parents were encouraged by the Ministry of Health’s support and their thoughts on the vaccine’s effectiveness in preventing the disease (35.1%). Some parents cited no specific reason (16.7%), whereas others were influenced by their physician’s advice (8.1%). A small proportion had other reasons for vaccination (1.6%).

**Figure 3:**
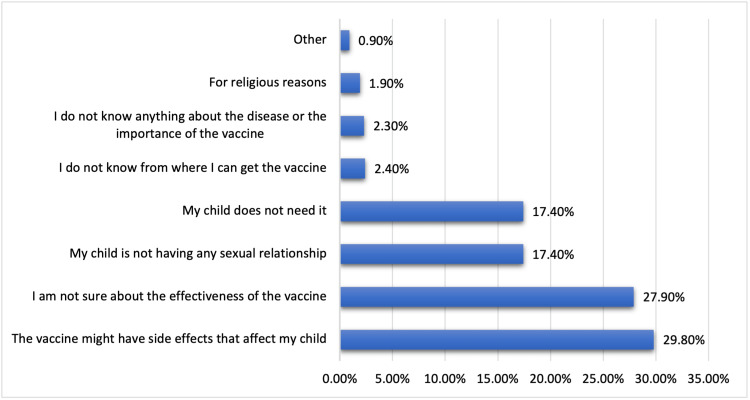
Factors preventing parents from vaccinating their child against human papillomavirus.

Figure 3 shows the factors preventing parents from vaccinating their children against HPV. Concerns about potential side effects affecting their child (29.8%) and uncertainty regarding the vaccine’s effectiveness (27.9%) were prominent factors. Some parents cited their child’s lack of sexual activity (17.4%) or the belief that their child does not need the vaccine (17.4%). A smaller percentage mentioned a lack of knowledge about the disease and vaccine importance (2.3%), and not knowing where to obtain the vaccine (2.4%). A few parents cited religious reasons (1.9%), whereas others had different concerns (0.9%).

Table 3 lists the significant associations between sociodemographic factors and general knowledge about HPV. Notably, gender played a role, with females showing higher knowledge (n = 171) than males (n = 81), which was a significant difference (p = 0.007). Age was influential, with older individuals exhibiting higher knowledge, particularly those 41-50 years of age (n = 99), and a weaker association among those <30 years of age (n = 29) (p < 0.001). Educational status also mattered, as those with a bachelor’s degree or higher had greater knowledge (n = 190) compared to those with up to a high school education (n = 62) (p=0.041). Employment status was also significant, with employed individuals displaying higher knowledge (n = 138) (p = 0.047). Additionally, the number of children influenced knowledge, with those having three to four children displaying higher knowledge (n = 91) compared to those with >7 children (n = 17) (p = 0.025). Notably, having heard of HPV was strongly associated with higher knowledge (n = 165) compared to those who were not sure (n = 22) or had not heard of it (n = 65) (p < 0.001).

**Table 3:** Associations between sociodemographic features and general knowledge about the human papillomavirus (HPV). *: P-value is calculated with Fisher’s exact test. No/Lower knowledge includes poor and fair.

			General knowledge about HPV		P-value*
			lower/No knowledge	Higher knowledge	
Gender	Female	n	68	171	0.007
		%	28.4%	71.6%	
	Male	n	60	81	
		%	42.5%	57.5%	
Age (years)	18–30	n	13	29	<0.001
		%	30.9%	69.1%	
	31–40	n	24	84	
		%	22.2%	77.8%	
	41–50	n	48	99	
		%	32.6%	67.4%	
	>50	n	43	40	
		%	51.8%	48.2%	
Educational status	Up to high school	n	45	62	0.041
		%	42.0%	58.0%	
	Bachelor’s or higher	n	84	190	
		%	30.6%	69.4%	
Occupation	Employed	n	62	138	0.047
		%	31.0%	69.0%	
	Freelancer	n	4	12	
		%	25.0%	75.0%	
	Housewife	n	11	7	
		%	61.1%	38.9%	
	Retired	n	26	32	
		%	44.8%	55.2%	
	Student	n	3	7	
		%	30.0%	70.0%	
	Unemployed	n	22	56	
		%	28.2%	71.8%	
Work field	Healthcare sector	n	9	25	0.448
		%	26.4%	73.6%	
	Other sectors	n	119	227	
		%	34.3%	65.7%	
Marital status	Married	n	126	244	0.756
		%	34.0%	66.0%	
	Widow/Divorced	n	3	8	
		%	27.2%	72.8%	
Number of children	1–2	n	30	73	0.025
		%	29.1%	60.9%	
	3–4	n	33	91	
		%	26.6%	73.4%	
	5–6	n	50	71	
		%	41.3%	58.7%	
	>7	n	15	17	
		%	46.8%	53.2%	
Have you heard of HPV?	No	n	97	65	<0.001
		%	59.8%	40.2%	
	Not sure	n	19	22	
		%	46.3%	53.7%	
	Yes	n	12	165	
		%	9.2%	92.8%	

Table 4 shows the associations between sociodemographic features and acceptance of the HPV vaccine. Gender played a vital role, with females exhibiting higher acceptance (n = 166) compared to males (n = 72), which was a highly significant difference (p < 0.001). Age also had a substantial impact, as older participants demonstrated greater acceptance, particularly those 41-50 of age (n = 96), whereas participants 18-30 years of age had the lowest rate of acceptance (n = 30) (p < 0.001). Educational status also mattered, with those holding a bachelor’s degree or higher displaying higher acceptance (n = 180) compared with those with up to a high school education (n = 58) (p = 0.045). Employment status was significantly associated with acceptance of the HPV vaccine, with employed individuals showing higher acceptance (n = 132) (p < 0.001). The number of children also influenced acceptance, as those with three to four children displayed higher acceptance (n = 86) (p = 0.015). Importantly, having heard of HPV was strongly associated with higher acceptance (n = 154) compared to those who were not sure (n = 24) or had not heard of it (n = 60) (p < 0.001).

**Table 4:** Associations between sociodemographic features and acceptance of the human papillomavirus (HPV) vaccine. *: P-value is calculated with Fisher’s exact test.

			Acceptance of HPV vaccine		P-value*
			No/Poor acceptance	Good acceptance	
Gender	Female	n	73	166	<0.001
		%	30.5%	69.5%	
	Male	n	69	72	
		%	48.9%	51.1%	
Age (years)	18–30	n	12	30	<0.001
		%	28.5%	71.5%	
	31–40	n	31	77	
		%	28.8%	71.2%	
	41–50	n	51	96	
		%	34.6%	65.4%	
	>50	n	48	35	
		%	57.8%	42.2%	
Educational status	Up to high school	n	49	58	0.045
		%	45.7%	54.3%	
	Bachelor’s or higher	n	94	180	
		%	34.3%	65.7%	
Occupation	Employed	n	68	132	<0.001
		%	34%	66%	
	Freelancer	n	8	8	
		%	50%	50%	
	Housewife	n	13	5	
		%	72.2%	27.8%	
	Retired	n	32	26	
		%	55.1%	44.9%	
	Student	n	4	6	
		%	40%	60%	
	Unemployed	n	17	61	
		%	21.7%	78.3%	
Work field	Healthcare sector	n	9	25	0.196
		%	26.4%	73.6%	
	Other sectors	n	133	213	
		%	36.5%	63.5%	
Marital status	Married	n	139	231	1.000
		%	37.5%	62.5%	
	Widow/Divorced	n	4	7	
		%	36.3%	63.7%	
Number of children	1–2	n	33	70	0.015
		%	32.0%	68.0%	
	3–4	n	38	86	
		%	30.6%	69.4%	
	5–6	n	53	68	
		%	43.8%	56.2%	
	>7	n	18	14	
		%	56.2%	43.8%	
Have you heard of HPV?	No	n	102	60	<0.001
		%	62.9%	37.1%	
	Not sure	n	17	24	
		%	41.4%	58.6%	
	Yes	n	23	154	
		%	12.9%	87.1%	

## Discussion

HPV is a common sexually transmitted virus associated with conditions such as skin warts and cervical cancer. Although many individuals clear the infection, some face persistent risks. Cervical cancer, linked to certain types of HPV, is a major health concern globally and in Saudi Arabia [14]. Our study aimed to assess the parental awareness and acceptance of the HPV vaccine for their children and identify the factors influencing their acceptance or refusal. Our study highlights important findings, which will be discussed in the context of existing medical literature.

Our study population is reflective of the Eastern Region of Saudi Arabia, where the majority of participants were female (62.8%) and married (97.3%). These results align with the cultural norms and expectations within the region, where mothers often take on a prominent role in childcare [15,16]. The high educational attainment (72.2% with a bachelor’s degree or above) we observed was also encouraging, suggesting a well-educated sample.

However, knowledge levels about HPV were modest, with a mean knowledge score of 3.00 out of 11. This result indicated a significant knowledge gap among parents in the region. These findings are in line with previous research in Saudi Arabia, which also reported insufficient knowledge about HPV and the vaccine among parents. For example, Alkalash et al. found that only 32.9% of their study participants had heard about HPV, and their knowledge scores were similarly low [13].

The vast majority of parents (62.9%) noted that their doctors discussed the HPV vaccine, raising concerns about the role of healthcare providers in vaccine promotion. Previous Saudi research by Osaghae et al. emphasized the significance of healthcare provider recommendations and confidence in counseling hesitant parents, highlighting a need for enhanced communication about the importance of the HPV vaccine [17].

Furthermore, a significant proportion (67.1%) of parents indicated that their children had not received the HPV vaccine, reflecting a concerningly low uptake despite the vaccine’s potential to prevent cancer. These results align with Alghamdi et al.’s study in Saudi Arabia, which showed that the majority of parents exhibited positive knowledge, attitudes, and practices regarding vaccination, which were influenced by sociodemographic factors. Nonetheless, addressing vaccination hesitancy by further targeting the identified contributing factors is warranted [18].

Approximately 41.1% of parents expressed willingness to have their child vaccinated by age 12, which is a promising prospect, as HPV vaccination is recommended at 11-12 years, indicating a receptive target group. Understanding the factors that influence this willingness to vaccinate at the recommended age can provide insights for interventions to boost vaccination rates [19].

Various factors influence parents’ decision to vaccinate their children against HPV. Notably, 38.5% cited the support of the Ministry of Health and 35.1% emphasized belief in the vaccine’s effectiveness as the key drivers for acceptance, aligning with similar findings in international studies, such as Kolek et al.’s work in Kenya [20].

However, the fact that a significant percentage of parents cited “no specific reason” (16.7%) for vaccinating their children suggests a lack of awareness or passive attitudes toward vaccination. Improving communication and education campaigns to emphasize the safety and effectiveness of the HPV vaccine may address this group.

Various factors also prevented parents from vaccinating their children. Concerns about potential side effects affecting their child (29.8%) and uncertainty regarding the vaccine’s effectiveness (27.9%) were prominent barriers. These concerns echo findings from various studies, including a review by Zheng et al., which highlighted concerns about safety as a consistent barrier to HPV vaccination [21]. Notably, a considerable percentage (17.4%) cited their child’s lack of sexual activity as a reason not to vaccinate. This misconception is an essential point to address, as HPV vaccination is most effective when administered before sexual debut. Religious reasons (1.9%) were also cited as a barrier. Therefore, it is crucial to engage religious leaders and scholars to clarify that the vaccine is compatible with Islamic values. A study by Hamdi et al. demonstrated Islamic teachings and scholars’ perspectives to understand the cultural tensions around sexuality, shedding light on barriers to vaccine acceptance [22].

There were significant associations between sociodemographic factors and general knowledge about HPV. Gender, age, educational status, employment status, and number of children were linked to HPV knowledge levels. Women exhibited higher knowledge compared to men. Older individuals and those with higher education had better knowledge, as did employed individuals and those with three to four children. Additionally, having heard of HPV was strongly associated with increased knowledge. These findings mirror previous studies both from Saudi Arabia and internationally [23].

This study did have some limitations. First, the study design was an online questionnaire; more accurate results could have been obtained with a physical form. Second, 62.8% of the participants were female (i.e., mothers); having an equal number of male and female participants in future studies is recommended for better representation of the results.

## Conclusions

This study reveals the insufficient level of knowledge about the HPV vaccine among both male and female Saudi parents in the Eastern Region of Saudi Arabia. This study highlights the need for targeted awareness campaigns to improve HPV vaccine knowledge and acceptance, especially among males and younger individuals. Healthcare providers should actively recommend the vaccine, address concerns about its side effects, and emphasize its effectiveness. Collaboration with religious leaders and educators is crucial to bridge knowledge gaps and reduce HPV-related health disparities. This study informs public health strategies to enhance vaccine coverage and reduce HPV-related diseases.
